# Gigification of Nursing Services in the United States, the United Kingdom, and Canada: Legal, Ethical, and Policy Implications

**DOI:** 10.1177/15271544261441590

**Published:** 2026-04-21

**Authors:** Jonathan Bayuo, Prince Kyei Baffour, Elisha Baafi Oduro, Emmanuel Akwasi Marfo

**Affiliations:** 1 School of Nursing and Midwifery, 7932 University of Southern Queensland, Ipswich, Australia; 2 School of Health, 4467 Leeds Beckett University, Leeds, UK; 3 School of Nursing, 12265 University of Maryland, Baltimore, Maryland, United States; 4 Department of Community Health Sciences, Cumming School of Medicine, University of Calgary, Calgary, Canada; 5 Faculty of Nursing, 3158 University of Alberta, Edmonton, Canada

**Keywords:** ethics, gig work, labor classification, nursing, platform work

## Abstract

**Objective:**

To examine the legal, ethical, and policy issues associated with gig work in nursing across the United States, the United Kingdom, and Canada.

**Methods:**

Narrative review and cross-country comparative analysis.

**Results:**

Sixteen studies were included. While the term “gig work” is not generally applied to nursing in both the United Kingdom and Canada, the availability of flexible, temporary, short-term work is common, manifesting primarily through either bank nursing or agency nursing mediated by various digital platforms. The phenomenon of internal banking was observed across all three countries. Despite the increasing trend of gig work, legal ambiguities exist regarding the classification of nurses as employees or independent contractors which has significant ramifications for liability and accountability. With the short-term nature of gig work, patient safety concerns also exist, particularly for nurses navigating new healthcare contexts. Compounding these challenges, many gig platforms lack standardized mechanisms to verify nurses’ credentials or enforce compliance with scope-of-practice regulations. Ethically, this regulatory vacuum perpetuates systemic inequities, as gig nurses may face substandard wages, exclusion from benefits, and exploitative contractual terms.

**Conclusion:**

While gig work offers nurses unprecedented autonomy and flexibility, its unchecked growth risks normalizing precarious labor conditions, eroding workplace protections, and raising patient safety concerns. To sustainably integrate the gig model, legislators must close classification loopholes. Healthcare institutions should implement registries for vetted gig workers and enforce standardized onboarding protocols to maintain care quality. Simultaneously, gig platforms require regulatory oversight to mandate real-time credential verification, wage guarantees, and scope-of-practice safeguards.

## Background

Gig nursing involves the use of a flexible nursing workforce for on-demand staffing, leveraging technology like mobile apps for scheduling, shifting, and payment. Gig nursing facilitates a work arrangement where nurses take on temporary, flexible assignments, often facilitated through digital platforms or digital staffing agencies (Singh et al., 2024). This approach allows nurses to work on a per diem or freelance basis, picking up shifts that vary in length, while healthcare facilities gain access to temporary staff to fill immediate or short-term needs (Lien, 2025). Comparatively, the gig nursing model differs from the traditional travel nursing in its app-managed, on-demand nature, and the use of independent contractor workforces.

The gig nursing model is part of the broader gig economy, where workers engage in short-term, task-based, or freelance jobs rather than traditional full-time employment. Gig nursing is like agency nursing, bank nursing, freelance work, or locum tenens albeit nuanced differences exist regarding who acts as the intermediary. For instance, for nurses who are employed by an agency, the agency acts as an intermediary between the nurse and healthcare facilities (Ruyter, 2007). That is, the agency handles the administrative tasks, such as payroll and scheduling, and provides nurses with temporary assignments at hospitals, clinics, or care homes (Simpson & Simpson, 2019). The gig nursing model is also akin to freelance work, where nurses use digital platforms or apps to find and accept individual shifts or short-term assignments directly. Also, whereas locum assignments can last for few weeks to several months to fill a temporary vacancy in a healthcare facility (Simpson & Simpson, 2019), gig nursing usually ranges from a single shift to few days, often facilitated through digital platforms or apps (Naghieh, 2020). Put together, gig nursing just like agency and bank nursing forms part of the broader temporary staffing arrangements. This article operationalizes the term “gig nursing” as a model which involves registered nurses work on a flexible, on-demand, and temporary basis, often facilitated by a third-party intermediary (Alanezi & Alanzi, 2020). Given that the term “gig nursing” is only an emerging terminology, we acknowledge that it may not be consistently applied in some settings. Thus, other forms of flexible, temporary models such as agency and bank nursing are presented and contrasted where possible.

Gig nursing promises flexibility, access to a diverse array of healthcare environments, short-term assignments, and platform-based engagement (connect with healthcare facilities via online platforms or apps). Gig nursing works just like Uber: the company (healthcare setting) and the driver (available nurse) are instantly matched based on available openings, leading to a filled opening for that shift following which the nurse is paid promptly for their service (Lien, 2024). This quick fix approach has been touted as extremely advantageous for both nurses who can decide which days to work and healthcare facilities who are able to rapidly fill staffing gaps. That is, the gig nursing model leverages the extensive on-demand workforce, providing nurses with greater autonomy to decide when and where they work, as well as how quickly they receive payment (Lien, 2023; Singh et al., 2024).

The rise of the gig economy in nursing, however, presents a complex blend of challenges and opportunities that can affect healthcare delivery, workforce stability, and patient outcomes. That is, while the gig nursing model offers some benefits, such as increased flexibility and autonomy for nurses to select shifts and work environments that align with their personal and professional needs, it also brings to light critical legal and ethical issues that have been largely overlooked (Pankaj & Jha, 2024; Singh et al., 2024). These include challenges related to job security, benefits, and continuity of care, necessitating a careful balance between flexibility and responsibility. Additionally, concerns about liability, insurance, and compliance with labor laws complicate the gig nursing landscape, making it essential for nurses to be informed about these issues before participating in gig work. Despite this critical gap and the gradual uptake of the gig nursing model post-COVID-19, the literature available on the subject remains extremely scanty raising concerns how nurses can effectively navigate the gig nursing landscape in developed countries such as the United States, the United Kingdom, and Canada. Theoretically, the study acknowledges that while labor and healthcare systems vary across the United Kingdom, the United States, and Canada, it may be possible to ascertain shared experiences of the same emerging phenomenon (gig nursing) which may have greater utility and lessons for other settings rather than just a single country analysis. It is against this background that this article employs a narrative review and cross-country comparative analysis approach to shed light on the legal and ethical issues associated with the gig model in nursing in the United States, the United Kingdom, and Canada. A secondary goal is to articulate policy implications of the gig model in the nursing profession and stimulate further discourse within the nursing community.

## Design

Considering the complex and broad nature of the gig economy through a cross-country comparative lens, the narrative review approach was employed (Ferrari, 2015). Narrative reviews offer a flexible and adaptable approach for broad, complex topics that require nuanced descriptions and interpretations as required in this current study (Ferrari, 2015). However, given the lack of standardized reporting guidelines for narrative reviews, applicable sections of the PRISMA Extension Guidelines for Scoping Reviews were incorporated into this study to enhance transparency and rigor (Tricco et al., 2018). Though a protocol was developed to guide this study based on existing best practices (Khalil et al., 2021; Peters et al., 2022), it was not published.

### Information Sources, Eligibility Criteria, and Search Strategy

PubMed, EMBASE, CINAHL, Web of Science, Scopus, and Google Scholar were searched extensively for studies reporting on gig work in nursing across three countries: the United States, the United Kingdom, and Canada. These countries were selected due to emerging evidence regarding the use of the gig model in the nursing profession. Following full-text retrievals, the reference sections were manually searched for potential papers. Considering narrative reviews permit the inclusion of gray literature sources, we also searched MedNar, OpenGrey, Trove, Agency for Healthcare Research and Quality, WorldWideScience, the Internet Archive, and BASE. As inclusion criteria, all databases were searched independently by two authors for relevant US, UK, and Canadian studies/reports published from 2005 to 2025. Thus, studies published before 2005 were excluded. Also, studies from other countries other than the United States, the United Kingdom, and Canada were excluded. Potential sources of evidence were considered for inclusion if they were reported in English. Language restriction was applied due to make the review more feasible for a small team with limited linguistic capacity (only documents in English were considered). Evidence sources reporting on gig work in nursing, agency nursing, temporary work, or on-demand nursing were considered for inclusion. No other limitations were applied in terms of study design considering the narrative review nature of the study. The searches were undertaken on February 10, 2025 and repeated on October 1, 2025.

The search terms used were: “gig economy” OR “platform economy” OR “gig work” OR “platform work” OR “freelance” OR “short-term” OR “temp” OR “temporary” AND “nursing” OR “nurse.” As part of the review protocol, the search terms were piloted with the assistance of our faculty librarian who further assisted the team to refine the terms and tailor these to each database. Both Medical Subject Headings (MeSH) and free-text search strategies were used. All screening and evaluation were undertaken independently by two authors. In case of disagreements, a third independent team member was invited.

### Selection of Sources of Evidence

**Figure 1.**
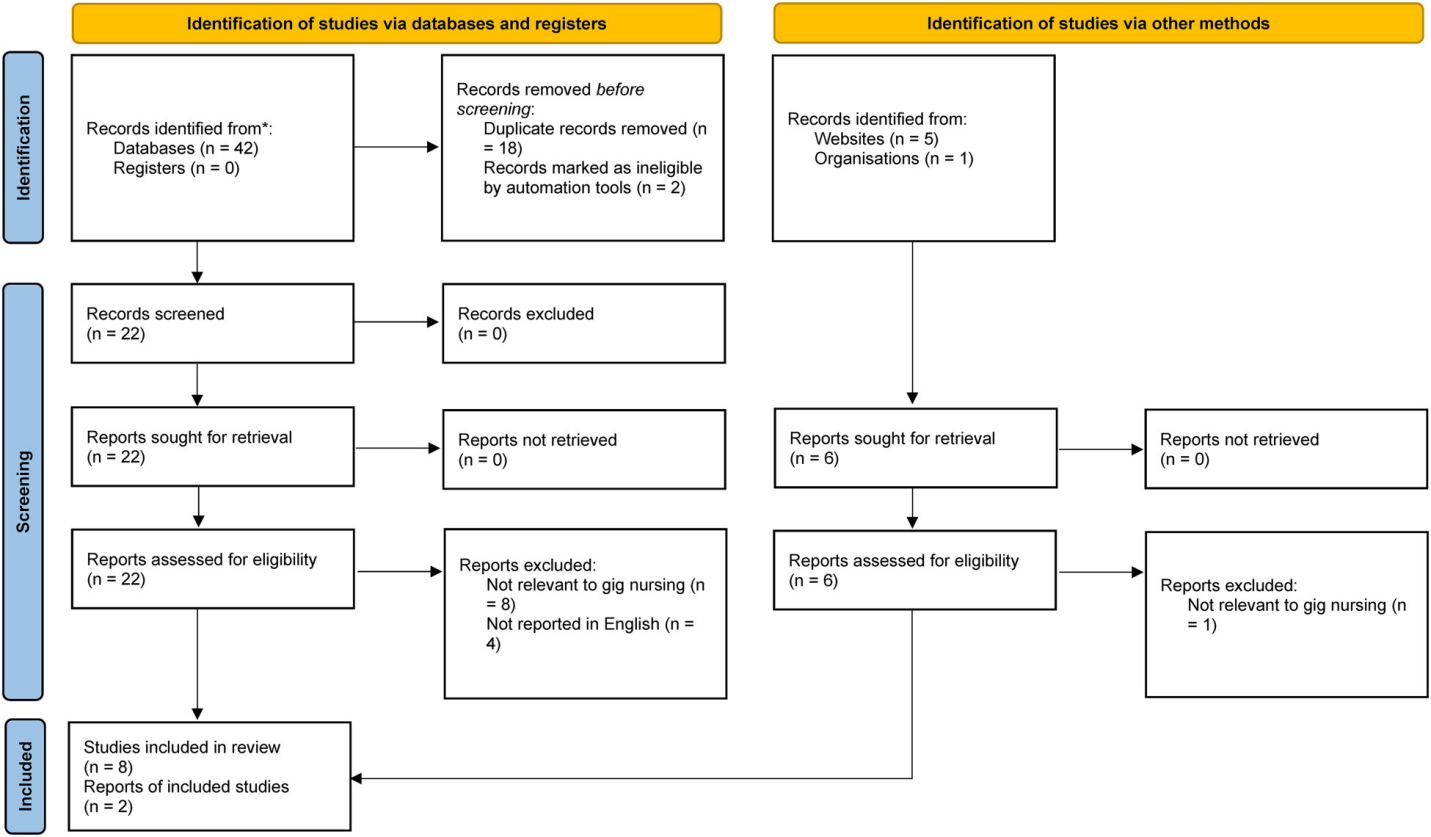
PRISMA flowchart for study selection.

Following extensive database and gray literature searches, all identified primary studies, gray literature, and reports were pooled to EndNote X9 for initial screening and de-duplication. Title and abstract screening were undertaken to ensure the sources of evidence focused on gig work in nursing across the United States, the United Kingdom, and Canada. At this stage, sources of evidence that did not report findings regarding gig work in nursing were excluded and the remaining sources progressed to the full-text screening stage. During that stage, full versions of the sources of evidence were retrieved with the assistance of the faculty librarian. The screening process was completed by two authors independently with ongoing wider team consultation. The selection process is detailed in a PRISMA flowchart (Figure 1). While critical/quality appraisal is not a requirement for narrative reviews, included gray literature was critical appraised using the AACODS (Authority, Accuracy, Coverage, Objectivity, Date, and Significance) checklist to ensure relevance and credibility to the phenomenon under exploration (Tyndall, 2010). Gray literature with a total number of “yes” scored as ≥4 on the AACODS checklist was considered credible for the purpose of this study.

### Data Charting and Synthesis

All studies and gray literature sources were reviewed and read severally to ascertain their focus. For each included evidence source, we extracted standard information such as authors, publication year, setting, type of study, findings, and findings relating to gig work in nursing (see Table 1). Coding was undertaken by identifying and highlighting the aspect of gig work being reported across each source of evidence in form of codes by two team members. The codes were subsequently grouped and reformulated into categories which formed the basis of undertaking a narrative synthesis. Following this, the findings for each included country were reviewed to ascertain how they diverge or converged from each other which helped to undertake the cross-country comparative analysis. Throughout the synthesis, all disagreements were resolved via discussion and the engagement of an independent member of the team.

**Table 1.** Evaluation of Gray Literature Sources.

Author(s)	Authority	Accuracy	Coverage	Objectivity	Date	Significance
Armstrong (2017)	Yes	Unclear	Unclear	Yes	Yes	Yes
Freyer (2025)	Yes	Yes	Unclear	Yes	Yes	Yes
Lam and Triandafyllidou (2022)	Yes	Yes	Yes	Yes	Yes	Yes
Levy (2016)	Yes	Yes	Yes	Yes	Yes	Yes
Vicks (2022)	Yes	Yes	Unclear	Yes	Yes	Yes

## Results

Sixteen sources of evidence (see Table 2) comprising of seven peer-reviewed primary studies (Alameddine et al., 2006; Bourbonniere et al., 2006; Drost et al., 2024; Lien, 2023; Ruyter, 2007; Seo & Spetz, 2013; Tailby, 2005); five gray literature materials (Armstrong, 2017; Freyer, 2025; Lam & Triandafyllidou, 2022; Levy, 2016; Vicks, 2022); one policy document (Morgan, 2022), one peer-reviewed literature review (Yang et al., 2023), one book chapter (Naghieh, 2020), and one doctoral thesis (Lien, 2024). The publication year ranged from 2005 to 2025. All the included gray literature materials scored ≥4 number of “yes.” Two categories emerged: (1) country snapshots of gig work in nursing and (2) legal and ethical issues associated with gig nursing.

**Table 2.** Data Extraction.

Authors/ Year/ Setting	Objective	Details	Key Findings
Alameddine et al., (2006) Canada	To examine the changes in the absolute and relative size of the nursing workforce by sector/subsectors in Ontario, Canada	All nurses registered with the Ontario College of Nurses over the 11 years from 1993 to 2003 were categorized as Active, Eligible or Not Eligible. Active nurses were then categorized by sector (Hospital, Community, Other) and subsector	The decline in Active and Eligible nurses was particularly pronounced for younger workers. Both the absolute number and proportion of nurses working in the hospital subsector has dropped. In the community sector, growth was evident in the use of nurses as case managers (in the CCAC subsector), community agencies, and community mental health (representing a shift from hospital-based workers). However, the steady growth in the number and proportion of nurses working in home care agencies was reversed in 1999, with this subsector shedding 19% of its nurses by 2003
Armstrong (2017) UK	To report on the gig economy trial app for nurses in the National Health Service (NHS)	Gray literature	The app would work alongside a system of staff banks to increase flexible working and help the NHS to be much better at supporting [nurses] with their own caring responsibilities
Bourbonniere et al. (2006) USA	To examine the extent to which nursing homes rely on the use of contracted licensed staff, factors associated with this staffing practice, and the resultant effect on the quality of resident care has received little public attention	Online Survey Certification and Reporting System database with the Area Resource File from 1992 through 2002	Since 1997, the proportion of facilities using 5 percent or more contract licensed staff more than tripled. Use of contract nurses was associated with more deficiency citations, characteristics of poorer facilities, and tight labor markets. Nursing homes increasingly rely on contract nurses
Ruyter (2007) UK	To explores the reasons why nurses in the United Kingdom work on an agency basis	A survey of nurses in two nursing agencies was conducted in 2005	While nurses who also have a permanent job are more likely to report pecuniary factors as influencing the decision to work through agencies, nurses who solely work agency are more likely to emphasize work–life balance issues and escaping “office politics” as key factors. Importantly, the findings point to the continued appeal of agency work to exit the nursing workplace, suggesting that government reforms to increase the appeal of nursing as a profession have only partially addressed the concerns of nurses
Drost et al. (2024) Canada	To examine the trends in the count and share of nurses working for employment agencies	2011 to 2021 regulatory college data on all registered nurses (RNs) and registered practical nurses (RPNs) in the province of Ontario, Canada	The prevalence of RNs and RPNs reporting agency employment was relatively stable from 2011 to 2019 and decreased slightly in 2020 and 2021. However, there was a small increase in transitions from nonagency employment to working at an agency job
Freyer (2025) USA	To discuss on-demand nursing jobs	Report/gray literature	For nurses, these online platforms allow for much-prized flexibility and greater work–life balance. They work when they want, at the facilities they choose. They can schedule a shift weeks in advance or moments before it begins. For healthcare facilities, the technology enables them to respond to fluctuations in patient demand, including unforeseen surges and staff illness, while drawing from the nursing workforce in their communities. This approach can be more cost-effective for them than using travel nursing or traditional per diem staffing agencies, and they don't have to commit to a certain number of shifts
Lam and Triandafyllidou (2022) Canada	To discuss the use of gig platforms by immigrant care workers	Gray literature	Digital care platforms are different than casual, one-off gig platforms like ride-hailing or food delivery. These latter forms of work are nonrelational, since there is neither a need nor a possibility of relationships forming between customers and service providers
Levy (2016) USA	To examine the impact of gig economy on legal classification of health care workers	Gray literature	This new business model combines attributes of both Uber and Handy and is an example of why a third worker classification model may be needed, one that requires workers to receive certain benefits but which characterizes the workers as independent contractors for tax purposes. Healthcare providers should be aware of the possibility that future Congressional legislation in this area might create savings by eliminating the need for health care companies to use professional employer organizations or other outsourcing providers as new, innovative business models are developed in the future
Lien (2023) USA	To explore how app platforms have reconfigured the care landscape of US nursing homes	12 months of ethnographic research collected between June 2020 and May 2021 at a 90-bed nursing home located in a Midwestern American state	The findings indicate that gig services (1) offer gig nurses monetary incentives at the expense of exaggerating the budget deficit of nursing homes, (2) increase gig nurses’ working autonomy while creating a loophole in managerial oversight, (3) lead to an uneven distribution of duties between gig nurses and nursing home staff, which demoralizes the latter. Taken together, gig services, while empowering the gig nurses, have resulted in the precarisation of nursing home staffing and care environments
Lien (2024) USA	To examines the organization, distribution, and ramifications of gig care work	Archival research, real-time observations of gig platforms, fieldwork in nursing homes, and interviews with nurses, managers, and care recipients	Nurses are turning to gig work as an act of self-preservation in response to the harms experienced in care work. By framing nurses’ transitions to gig work as a form of self-preservation, this project also brings to light the unequal distribution of resources within the US capitalist healthcare system, where worker exploitation is not an anomaly but a fundamental element of the nursing home industry
Morgan (2022) UK	To identify why ministers are unlikely to take meaningful, long-term action to address NHS workforce shortages	Policy document	Some possible solutions to help overcome workforce shortages include having greater transparency in workforce forecasts, establishing an independent workforce-planning organization, and accepting the need for international recruitment
Naghieh (2020) UK	To introduce the contribution of the gig economy to the declining power of the medical profession	Book chapter	The gig economy disaggregates medical work into isolated on-demand microtasks on digital platforms. This has implications for the status and power of the medical profession, the healthcare provider–patient relationship, and interprofessional boundaries in healthcare
Seo and Spetz (2013) California, USA	To examine the trend of agency nursing during the years of RN shortage and the factors that affect demand for temporary personnel	RN Data from California's Office of Statewide Health Planning and Development (OSHPD) from 1993 through 2002	A significant trend toward higher use of agency nurses during the years of labor shortage was observed. Agency nurse hours as a proportion of total RN hours also grew indicating that hospitals use temporary contract nurses to deal with RN shortages
Tailby (2005) UK	To explore the forms of temporary employment in nursing, the reasons nurses give for taking temporary or agency work, as a main job or a second job, and their experiences in such employment	Case study involving four nursing support staff and 28 registered nurses (in grades between D and G of the national pay and grading structure)	Bank nursing had supplied an easier means of balancing paid work and child-care than the part-time NHS employment that had been available to them. Bank-only nursing allowed nurses to specify their availability for work, but it had disadvantages. In the past it had meant exclusion from the employer beneﬁts that accrued only to standard employees—sick pay, holiday pay, access to the NHS pension scheme and to training. There had been some improvement since the late 1990s, not least because Trust managers had been obliged to respond to the competition for casual staff from private sector agencies. Even as a repeat worker, however, the bank nurse was a temporary addition to the ward's regular team rather than a fully-ﬂedged member of it. There was less opportunity to reap the social rewards of work, or to assert claims for equal treatment in respect of the allocation of work tasks and opportunities for skills upgrading. Additional shifts were remunerated at overtime, bank or agency rates. Full-time staff had more access to premium rates than nurses working part time. Additional shifts were worked to increase earnings, although some nurses had additional or complementary objectives, and while pay was an important inﬂuence on nurses’ choices between overtime, bank and agency nursing, it was not the exclusive inﬂuence. Shifts were often “booked” informally on the ward, and nurses could use the computer terminals on the wards to view the vacancies offered by the internal nurse bank and to book shifts that were convenient for them. Bank nursing within the Trust meant a familiar environment and nurses had some choice in the wards on which they would work. This was important because it reduced the stress of temping and was a principal reason why many nurses preferred bank to agency nursing. Agency work was seen to offer higher pay but few other material rewards superior to NHS open-ended employment
Vicks (2022) USA	To examine how the big tech industry is making the terrible health care system worse	Grey literature	The ultimate problem with this lean philosophy is not just that it's inherently vulnerable to unpredictable events like a pandemic — though that is a serious flaw, as the last two years have demonstrated. The ultimate problem is that it treats workers and patients as little more than inputs in a system designed to generate profit.
Yang et al. (2023) USA	To clarify the controversies and challenges of nurse-on-demand models/platforms	Literature review	The primary concern is that independent contractors lose employee benefits and even worker protections, such as minimum wage guarantees, overtime pay, and discrimination safeguards. Misclassification of workers, especially in the healthcare sector, is a growing concern. When companies label workers as independent contractors, they can avoid providing benefits such as overtime pay and sick leave. The amalgamation of technology, gig economy, and health care via on-demand nursing platforms presents opportunities such as enhanced flexibility and reduced burnout potential. Despite these advantages, concerns around worker status, legal implications, and job equity arise, demanding careful consideration

CCAC: Community Care Access Centres.

### Category 1: Country Snapshots of Gig Work in Nursing

#### The US Picture

Although the current prevalence of nurses working through gig platforms in the United States remains undocumented, the use of temporary or contingent nurses has increased dramatically since the 1970s (Seo & Spetz, 2013). Significant growth occurred following managed care reforms, which imposed cost containment measures, and the shift from a nursing surplus to a shortage (Bourbonniere et al., 2006; Seo & Spetz, 2013). Despite the lack of documented data, some studies have highlighted an increasing number of nurses transitioning to the gig model to take on temporary positions in the United States (Bourbonniere et al., 2006; Lien, 2023, 2024). Also, available nursing demographic data suggest that gig nursing roles are predominantly filled by women and people of color, which raises significant concerns that gig nursing platforms may be exacerbating existing gender and racial inequities within the healthcare workforce (Yang et al., 2023).

Common platforms such as CareRev, Clipboard Health, ShiftKey, and Medely play the role of connecting nurses with temporary assignments. These platforms provide healthcare professionals with a convenient way to present their qualifications and shift preferences. They then connect these professionals with suitable facilities and open shifts (Yang et al., 2023). Offering greater flexibility than traditional travel nurse contracts, the platforms leverage local professionals to manage peak demand periods (Yang et al., 2023).

#### The UK Picture

While the term “gig work” (implying short-term, app-mediated, and flexible tasks) is not universally applied to nursing in the United Kingdom, the concept of flexible, temporary, on-demand nursing work is deeply embedded within the National Health Service (NHS) and private healthcare sector (Tailby, 2005). This manifests primarily through two established systems (bank nursing and agency nursing) and an emerging digital platform model which may reflects gig work (Tailby, 2005). Bank nursing is managed directly by individual NHS Trusts or private healthcare providers (Tailby, 2005). Nurses register with the Trust's internal “bank” and choose shifts (often via online portals or apps) as they become available, filling short-term vacancies due to sickness, leave, or increased demand (Tailby, 2005). For agency nursing, nurses register with commercial staffing agencies. These agencies contract with NHS Trusts and private providers to supply nurses for shifts. Agencies find and place nurses, handle payroll (including employer National Insurance contributions), and charge the Trust a fee significantly higher than the nurse's pay (Ruyter, 2007). The agency approach offers wider geographical flexibility and potentially higher pay rates than bank work (though the agency takes a large cut) (Tailby, 2005). However, it has been a major source of financial pressure for the NHS (Morgan, 2022).

In the United Kingdom, the NHS was expected to trial a gig economy app for nurses in early 2018; an approach which was criticized as an “uber model for the NHS” (Armstrong, 2017). Despite the criticism, technology platforms are emerging that connect nurses directly with available shifts across multiple NHS Trusts and private providers. These include Patchwork Health and Lantum which allow healthcare professionals to create profiles, set preferences/availability, and book shifts. The platform handles scheduling, timesheets, and payment. This approach most closely resembles the gig economy, and it offers maximum flexibility and user-friendly technology for the practitioners. Nurses are typically considered to be either self-employed (sole traders) or employed by the platform itself. Despite their gradual uptake, the actual number of nurses engaged on these platforms across the United Kingdom remains undocumented, just like the United States.

#### The Canadian Picture

Similar to the UK context, the term “gig nursing” is not generally applied to nursing in Canada though flexible, temporary, and often short-term nursing work represents a significant and growing component of the Canadian healthcare workforce (Alameddine et al., 2006; Drost et al., 2024). This model manifests primarily through agency nursing which remains the dominant model (Drost et al., 2024). Nurses register with private staffing agencies specializing in healthcare. Agencies contract with hospitals, long-term care (LTC) facilities, clinics, and public health units to fill temporary vacancies caused by illness, leave, vacancies, or surges in demand. Nurses are typically employed by the agency (W-2 equivalent in Canada) or contracted as independent contractors (T4A). The agency handles placement, scheduling (though nurses often have choice), payroll, and charges the healthcare facility a premium rate significantly higher than the nurse's pay. Assignments range from single shifts to longer-term contracts (weeks/months). Apart from the agency approach, the gig model in the Canadian context is also evident among part-time and permanent nursing staff.

Despite the extensive search undertaken, only anecdotal evidence was identified regarding the use of gig platforms among healthcare providers in Canada. For example, the Alberta Health Services (AHS) uses the Vocantas app for scheduling, which allows casual and part-time nurses to pick up shifts based on their own availability and preferences. At the same time, full-time permanent staff also engage with the platform to select overtime shifts when staffing shortages arise (internal banking). Apart from this, some available gig platforms are reportedly used by internationally nurses who are yet to complete the registered nurse credentialing/ licensing process (Lam & Triandafyllidou, 2022). These digital platforms such as Care offer the migrant nurses the opportunity to take up temporary work as healthcare aides or personal support workers as they navigate the nursing registration process in Canada (Lam & Triandafyllidou, 2022). While the apps offer an opportunity to work, it reinforces deskilling of highly trained professionals under the guise of healthcare employment. The authors further note that for immigrant healthcare professionals, care platforms may offer only a temporary reprieve (Lam & Triandafyllidou, 2022). They caution that while digital care platforms may enable immigrants to secure work aligned with their expertise or find meaningful employment, this access comes with significant drawbacks (Lam & Triandafyllidou, 2022). That is, workers on these platforms are often compelled to assume personal and professional risks, reinforcing exploitative power imbalances not only between workers and the platform but also between workers and care recipients (Lam & Triandafyllidou, 2022).

### Category 2: Legal and Ethical Issues Associated With Gig Nursing

#### Employment Status: Employees or Independent Contractors?

A key legal concern associated with gig work is the classification of its practitioners as independent contractors versus employees. This classification is critical considering its wide-ranging implications for the parties involved. Comparatively, the employee status confers a stable, long-term relationship with the employer with a higher job security albeit offering less flexibility whereas an independent contractor status reflects only a short-term with no job security (Yang et al., 2023). In the United States, individuals classified as employees are entitled to various legal protections and benefits, such as minimum wage, overtime pay, paid sick days, paid family leave, workers’ compensation, and unemployment insurance. In contrast, individuals classified as independent contractors operate as self-employed entities and not subject to the same legal protections and benefits as employees (Levy, 2016; Naghieh, 2020). Additionally, US labor laws confer various protections to employees, such as the right to a safe working environment, protection against discrimination, and the ability to organize and engage in collective bargaining. However, independent contractors, as self-employed individuals, are not covered by most labor laws in the United States.

Further to the above, independent contractors are responsible for paying their own taxes and do not receive any benefits, including over time and health insurance (Levy, 2016). In the US jurisdiction, employers are obligated to withhold income taxes, as well as Social Security and Medicare contributions, from their employees’ wages, paying these taxes on behalf of the employees. Conversely, independent contractors must handle their own tax payments, including self-employment taxes that cover Social Security and Medicare. The core vulnerability, therefore, lies in the loss of employee status: misclassified independent contractors forfeit critical benefits and fundamental worker protections, including guaranteed minimum wage, overtime pay, and safeguards against discrimination. For example, the CareRev gig platform has been reported to classify its nurses as independent contractors which shifts additional responsibilities and risks, such as obtaining malpractice insurance and meeting tax obligations, to nurses (Yang et al., 2023).

The Canadian jurisdiction also highlights a significant difference between employees and independent contractors, just like the US system (Fudge et al., 2003). Immigrant healthcare practitioners engaged via the gig platforms in Canada may usually be considered as independent contractors rather than employees (Lam & Triandafyllidou, 2022). While being classified as an independent contractor will allow the healthcare provider to work for multiple clients or companies, they are not entitled to benefit plans such as registered pension plans, group accident, health, and dental insurance plan (BDC, 2022).

In the United Kingdom, the description of a contractor encapsulates three distinct categories: self-employed (run their business on their own), employment status as a worker (have either a written or unwritten contract to undertake a service for payment), and employment status as an employee (employed by an agency and working on the basis of an employment contract) (UKGov, 2021). Comparatively, a “worker” status in the United Kingdom is associated with fewer rights than an employee. Since the digital apps used to connect gig nurses to available shift openings cannot be classified as agency, it could mean that UK nurses who take up platform roles may be classified under the “employment status as a worker” category (UKGov, 2021). Though workers under this category are entitled to certain employment rights such as national minimum wage, protection from unlawful discrimination and unlawful deductions and statutory redundancy pay are unavailable. Also, they are not protected from unfair dismissals, minimum notice if their employment will be ending; may not request for flexible work arrangements or time off for emergencies (UKGov, 2021).

#### Patient and Staff Safety

The potential impact of gig platforms on care quality and patient safety demands critical scrutiny (Yang et al., 2023). In fact, the gig model can impact patient safety if nurses are not adequately oriented or integrated into the healthcare team. The transient nature of gig work can disrupt continuity of care, as nurses frequently move between different healthcare settings, potentially affecting the quality and consistency of patient care (Yang et al., 2023). In the foundation ethnographic work by Lien (2023) which explored the impact of gig nursing on care homes in the US context, it was observed that gig nursing undermined facility efficiency, staff teamwork, and care standards—evidenced by links to increased catheter utilization and error rates. Lien's assessment is stark: gig nursing services have worsened the quality and availability of nursing positions, failing to alleviate the underlying problems (Lien, 2023). As Vicks (2022) note, the gig nursing model drops uninitiated strangers into already stressful situations and can have the paradoxical effect of making work harder for full-time staff trying to care for their patients while also bringing new staff up to speed.

In United States acute care settings, nurses working in units with more than 15% external temporary or agency nurses were more likely to report back injuries and greater levels of patient falls than the units that did not have the temporary nurses (Bae et al., 2010). Also, teamwork and communication have been observed to be particularly challenging on shifts with high levels of temporary staff, alongside increased medication errors in the United States (Pham et al., 2011). In the United Kingdom, increased use of temporary staffing (agency and hospital banks) was associated with a high risk of patients dying (Dall’Ora et al., 2020). Also, increasing the proportion of temporary nurse staff recruited either from external agencies or hospital banks was observed to be associated with higher rates of self-reported care left undone by nursing staff in the United Kingdom (Senek et al., 2020). In the Canadian context, a higher hospital death rate has been associated with higher temporary staffing, highlighting the adverse effect of the lack of continuity of care (Estabrooks et al., 2011). Together, these highlight critical patient and staff safety issues. While the studies highlighted here focus on temporary nurses obtained via external agencies or a hospital's own bank, gig nursing operates in a similar manner and may raise similar concerns. These may be related to the fact that the temporary nurses often lack familiarity with specific unit workflows, institutional protocols, and specialized equipment, which can compromise care standards compared to permanent, integrated staff (Yang et al., 2023). Critically, current platforms, driven primarily by cost-reduction objectives, fail to integrate essential quality metrics such as adverse event rates, patient satisfaction scores, or staff burnout levels. This oversight represents a significant systemic risk, prioritizing financial efficiency over demonstrable patient safety and care quality outcomes. Aside from patient safety concerns, issues about staff safety also remain a critical concern. The UK case study by Tailby (2005) highlighted instances of temporary nurses feeling a sense of exclusion from the team and occasional hostility experiences from others thought to be regular or permanent staff. Similarly, racial discrimination has been reported among nurses undertaking gig nursing jobs (Wells & Spilda, 2024).

#### Regulatory, Compliance, and Credentialing/ Licensure Issues

Further to the labor and liability issues associated with the gig model in nursing, it remains unclear how the apps/platforms that connect nurses to available shift openings ensure that they have the requisite training, clinical background, and are credentialed/ licensed within a practice scope. The platforms employed across the United States (Lien, 2023, 2024; Yang et al., 2023), the United Kingdom (Tailby, 2005), and Canada (Lam & Triandafyllidou, 2022) offer the nurses an opportunity to create a profile in which they can indicate their credentials and qualifications for matching purposes. However, it remains unclear who reviews and approves these professional profiles, ensuring that credentials and qualifications are accurate and up to date.

Ethically, the gig economy challenges traditional notions of professional responsibility and patient care. Healthcare professionals have an ethical obligation to provide consistent, high-quality care, regardless of their employment status. This requires a commitment to ongoing professional development and adherence to established standards of practice. Healthcare organizations and platforms facilitating gig work must also uphold ethical standards by ensuring fair treatment of workers and prioritizing patient safety and care quality (Yang et al., 2023). However, the gigification of healthcare impacts the responsibility and quality of care provided to patients (Lien, 2023, 2024). Gig workers may face difficulties in maintaining continuity of care, as they often work in different settings with varying protocols and patient populations. This can lead to inconsistencies in patient care and challenges in building trust and rapport with patients (Dall’Ora et al., 2020). Besides, gig nurses may have fewer opportunities for professional development and support compared to their full-time counterparts. This can affect their career growth and ability to stay updated with best practices. From both legal and ethical perspectives, the responsibility for patient outcomes can become blurred in gig work arrangements. Determining liability in cases of malpractice or negligence can be complex, as gig workers may not be covered by the same insurance policies as permanent staff. This necessitates a re-evaluation of frameworks to ensure that both patients and healthcare professionals are adequately protected.

#### Concerns Regarding Equity, Fair Compensation, and Potential Exploitation

Gig nurses may face issues related to equity, fair compensation and exploitation, as they might not receive the same pay or benefits as full-time staff (Lien, 2023, 2024; Yang et al., 2023). Evidence from the US context indicates gig nursing roles are predominantly filled by women and people of color (Yang et al., 2023). This may suggest that gig platforms may inadvertently—or structurally—rely on groups historically marginalized within the healthcare workforce. A parallel pattern emerges in Canada, where anecdotal and early empirical evidence indicate heavy reliance on unemployed immigrant healthcare workers (Lam & Triandafyllidou, 2022). In the United Kingdom, it has been reported that temporary workers experience discrimination from staff, organizations, and national bodies because of their working status, and in some instances, because of their ethnicity (HSSIB, 2024). Fear of losing future opportunities often leads to their inability to raise concerns (HSSIB, 2024). This convergence points to a critical equity concern: gig/temporary nursing platforms may be capitalizing on systemic barriers (e.g., credential recognition delays, discrimination in traditional hiring, and caregiving responsibilities often shouldered by women) that funnel vulnerable groups into contingent roles. Consequently, these workers face a double burden: exposure to platform-related precarity and the amplification of existing socioeconomic and racial inequities within the nursing profession.

Dynamic pay models, while potentially improving coverage for unpopular shifts, introduce significant systemic and ethical risks (Yang et al., 2023). Higher rates for undesirable shifts may attract staff, but risk triggering staffing imbalances elsewhere if nurses disproportionately pursue premium pay, leaving core shifts unexpectedly understaffed. This volatility directly threatens care continuity and patient safety. Furthermore, over-reliance on financial incentives risks corroding professional commitment, potentially diminishing intrinsic motivation for quality care and, in extreme cases, contributing to negligence (Yang et al., 2023). These models also disproportionately advantage nurses with financial security, enabling them to selectively pursue higher-paying shifts. Crucially, this approach fundamentally conflicts with nursing's core ethic of patient-centered care, creating a profound professional dilemma by placing financial gain in potential competition with optimal patient outcomes (Yang et al., 2023).

With employment status potentially classified as independent contractors, gig nurses will not be able to access several benefits (Lien, 2023, 2024; Ruyter, 2007; Spilda, 2024; Tailby, 2005; Yang et al., 2023). This lack of benefits raises ethical concerns about the equitable treatment of gig nurses compared to their traditionally employed counterparts. Now, it is unclear if existing gig apps obtain data regarding race/ethnicity and immigration status. The informal nature of the gig economy implies that gig workers are at a high risk of exploitation, insecurity, and exclusion (Pankaj & Jha, 2024). Besides, the classification as independent contractors means gig nurses are unable to form unions which can adversely impact their bargaining status. Just as Uber drivers can be laid off without any compensation, gig nurses do not have any binding contracts with the healthcare facilities they work for and can also be laid off or canceled without any form of compensation (“Little v BMI Chiltern Hospital", 2009). Besides, a recent extensive report regarding gig work in nursing highlighted that since the nurses are rated based on the feedback from the healthcare facility and algorithm determinations, gig nurses can be penalized if they canceled a shift due to sickness or had a conflict (Spilda, 2024). Potentially, this may mean that healthcare organizations may benefit more from the gigification of nursing, particularly in adverse situations, compared to nurses (Yang et al., 2023). That is, the apps also give healthcare facilities access to a larger labor pool, the ability to adjust rates as needed and an alternative to the more expensive option of using temporary staffing agencies.

## Discussion

The gigification of nursing services represents a significant shift in the healthcare and technological landscape, offering both opportunities and challenges. While the flexibility and autonomy of gig work can benefit nurses and systems, it also raises important legal and ethical questions. Addressing these issues requires a collaborative effort among policymakers, healthcare organizations, and gig platforms to develop frameworks that protect the rights and responsibilities of all stakeholders. By rethinking care, flexibility, and responsibility in the context of the gig economy, we can harness its potential while safeguarding the integrity and quality of healthcare services.

At the core of the gig model is the concern regarding classification of gig nurses as independent contractors rather than employees. Comparatively, the cross-country analyses suggest that nurses working in the gig economy may not be classified as employees due to the temporary nature of their assignments. However, some level of ambiguity seems to exist since the gig platforms do not make this explicit (Yang et al., 2023). Without clear legal criteria defining their employment status, there is a significant risk of misclassifying these healthcare professionals as independent contractors rather than employees. Such misclassification excludes them from essential benefits like health insurance, retirement plans, and paid leave, potentially perpetuating financial insecurity and diminished job satisfaction. Addressing this ambiguity in labor classification is therefore critical to ensuring equitable protections and sustainable career pathways for gig-based nursing professionals.

States in the United States have adopted divergent frameworks for distinguishing independent contractors from employees, with significant implications for healthcare labor markets. California exemplifies this regulatory complexity through Assembly Bill 5 (AB-5), enacted in 2019, which codified the stringent “ABC” test to determine worker classification (California Legislative Information, 2019). Under this law, most workers, including healthcare professionals, are presumed to be employees unless they meet three criteria: autonomy from employer control, work outside the employer's core business, and independent operation of a trade (California Legislative Information, 2019). While AB-5 exempts physicians, surgeons, dentists, and other select medical practitioners (Cal. Labor Code § 2783(b)), it notably excludes nurses, leaving them vulnerable to misclassification. In the gig economy, companies like Uber, Lyft, DoorDash, and other gig platforms have been heavily impacted. Under the ABC Test, many gig workers no longer qualify as independent contractors. This gap has already spurred litigation, including a pending class-action lawsuit against a nursing gig platform alleging unlawful designation of nurses as independent contractors (“Suttle v. Care.Stat! Inc., No. 21CV383162 Cal. Super. Ct.,”). Other states are now mirroring California's approach. Michigan recently proposed HB 4390 of 2023, which adopts a similar three-pronged assessment for contractor classification and imposes aggressive penalties for violations (“House Bill 4390 of 2023- Michigan Legislature,” 2023). The bill categorizes misclassification as a standalone offense under the state's Payment of Wages and Fringe Benefits Act, subjecting employers to civil fines of up to $10,000 per violation. Such measures signal a nationwide trend toward tightening labor standards, particularly in sectors like healthcare, where gig work risks eroding worker protections. These evolving laws highlight the growing tension between flexible labor models and the need to safeguard employee rights, urging employers to reassess compliance strategies amid heightened regulatory scrutiny.

For the United Kingdom, it is possible to glean from an existing case law to ascertain the status of nurses engaged in bank work. In *Little v BMI Chiltern Hospital*, Mr Little worked for the Respondent, BMI Chiltern Hospital Trust (“BMI”) as a “bank” theater porter for various periods between 15 October 1992 and 28 February 2008 (“Little v BMI Chiltern Hospital", 2009). Little averaged around 20 to 30 hours per week, although his hours did vary from time to time. Little's working arrangements were governed by written agreements confirming that he would work on an “as and when” basis. The agreements also confirmed that body mass index (BMI) would not guarantee to provide Little with work, and Little had the right to refuse any work he was offered, however if Little refused work on four consecutive occasions or was unavailable for work for four consecutive weeks, he would be removed from the bank. As part of the agreements governing the working relationship, *Little* had also signed a letter confirming there was no mutuality of obligation and each assignment, he was given was separate. BMI terminated Little's arrangement in early 2008 following which Little sought to bring an unfair dismissal claim. The Tribunal found that the lack of mutuality of obligations was evidenced by the fact Little had a choice of if and when he worked for BMI. Little appealed the decision but failed on the basis that his “bank” work lacked the mutuality of obligation. The implication of this precedent in the UK context is that the absence of an ongoing obligation on the employer to offer work and the worker to accept it is often fatal to establishing employee status. This highlights the legal vulnerability of individuals working under “as and when” arrangements, even if they work regularly for a single entity over time. Their rights depend heavily on the specific contractual structure.

In the Canadian context, there is no single test to determine if an employment relationship exists. Instead, legal precedents from tax and tort cases suggest several factors that are considered in combination to ascertain if such employment relationship is in existence. A summary of factors that suggest an employer–employee relationship highlighted by Canadian Nurses Protective Society (CNPS) include receiving fixed salary with routine deductions, unable to hire own helpers, cannot provide own equipment, required to report to a supervisor, employer control is exerted over the work through policies and procedures, bears no risks for the company's financial losses, and has no financial investment in the enterprise (CNPS, 2006).

Determining liability in cases of malpractice or negligence can be complex in the context of the gig nursing model (Lien, 2023, 2024). Gig nurses may need to carry their own malpractice insurance, and there can be ambiguity about who is responsible in the event of an error—the nurse, the app/platform, or the healthcare facility (Vicks, 2022; Yang et al., 2023). Across the United States, the United Kingdom, and Canada, the distinction between employees and contractors remains crucial when considering liability for workplace accidents or injuries (Pearce, 2018; Posner, 2021; Vicks, 2022; Yang et al., 2023). In the United States (Lien, 2023, 2024; Yang et al., 2023), the United Kingdom (Ruyter, 2007; Tailby, 2005), and Canada (BDC, 2022), employers are typically held accountable for any injuries or damages caused by their employees while performing work-related tasks. They are also required to carry workers’ compensation insurance, which provides coverage for employee injuries and protects both the employee and employer in the event of unforeseen accidents. In contrast, independent contractors, as self-employed individuals, are generally responsible for their own liability insurance across the United States (Lien, 2023, 2024; Yang et al., 2023), the United Kingdom (Ruyter, 2007; Tailby, 2005), and Canada (BDC, 2022; CNPS, 2006). Thus, if an independent contractor causes an accident, they are personally liable for any resulting damages across all three countries. For gig nurses classified as independent contractors, this represents a significant shift of burden, which traditionally should fall on their employers if they were classified as employees. In this way, the gig model represents a significant threat, particularly considering that several risks exist in healthcare and errors can potentially emerge.

Two key regulatory/credentialing concerns emerge from the operation of the gig approach. Firstly, it is unclear the controls in place to ensure only licensed nurses are linked to available shift slots, and secondly, it remains unclear how the app ensures that the assigned nurse has the requisite clinical background/experience to work in the assigned unit for the shift. With the short-term, shift-based nature of these gigs, there is a potential likelihood of lack of effective supervision, particularly if considered as an independent contract. From a compliance perspective, it can be daunting for a gig nurse to be fully aware of the policies and procedures employed in a healthcare facility before a shift commences (Yang et al., 2023). These can increase the chances of medical errors and pose significant risks to patient safety (Krzyzewski et al., 2024).

### Policy Implications

The nursing profession, like other sectors, faces transformative pressures from the gig economy and rapid technological advancements (Bayuo, 2024; Bayuo et al., 2023). To mitigate risks to workforce stability and patient care, regulators and healthcare institutions must proactively establish guardrails that balance flexibility with accountability. A notable innovation in this regard observed in the US context is the development of internal agency with flexible staffing arrangements within healthcare settings. This innovation mirrors practices currently in place with travel agencies across the United States. One study has reported that the approach led to the retention and rehiring of over 100 nurses in the first year and $12.7 million in cost savings (Bellon, 2023). In the United Kingdom, the NHS is also improving on its own staff banks to address their workforce shortages, reduce reliance on external agencies, and making renumeration for agency staff more stringent to cut spending (NHS, 2024). Though evidence regarding the impact of this arrangement is yet to be reported, it has been hypothesized that the NHS could free up to 480 million pounds by limiting the use of temporary staffing agencies (NHS, 2018). In Canada, a move toward banning the use of staffing agencies, creating regional or health authority floating pools, use of alternate staffing models (such as job-sharing or permanent part-time roles), and promoting flexible scheduling models has been proposed (HEC, 2025). Thus, it is evident that all three countries are working toward implementing flexible working schedules to improve retention. More research is needed to examine the impacts of these models to inform policy and drive change.

While gig nursing can temporarily alleviate staffing shortages, it should not supplant long-term strategies to address systemic issues like high nurse turnover. For example, healthcare organizations could retain permanent staff by competitive benefits, and career development opportunities, reducing reliance on transient gig workers (Lien, 2023, 2024; Spilda, 2024). Clear policies must also define minimum standards for engaging gig nurses, including mandatory onboarding aligned with facility protocols, verification of current licensure and clinical/speciality experience or competence, and adherence to safe staffing ratios under local regulations. Other standardized onboarding safeguards that should be integrated into gig work include unit/facility-specific medication and safety protocols to electronic health records (EHR) access and brief competency checks to ensure temporary nurses are well prepared for their roles, and to potentially minimize patient safety risks. Maintaining a registry of vetted gig nurses who have previously worked within a facility and hiring from that similar pool could streamline staffing processes, ensure continuity of care and reduce the risks associated with unfamiliar temporary workers, including patient safety risks.

Technological platforms connecting gig nurses to shifts demand rigorous oversight. Regulatory bodies should mandate that these apps integrate real-time verification of nurses’ credentials, scope-of-practice limitations, and continuing education status. Collaboration between app/platform developers and regulators is critical to standardize compliance with labor laws, enforce minimum wage guarantees, and prevent exploitative practices. Existing data gaps on the apps may also be remedied by including relevant indicators such as race/ethnicity and immigration status during the gig registration process. This approach may be helpful in facilitating monitoring of disparate impacts over time. Crucially, policymakers must modernize labor classifications to explicitly recognize gig nurses as employees entitled to traditional benefits, rather than independent contractors. Current legal frameworks lag behind technological innovation, creating ambiguity that leaves nurses vulnerable to misclassification and its consequences. Without updated statutes or judicial precedents, the rights of gig nurses remain precariously unprotected.

### Limitations

While the current study offers interesting insights into gig nursing, some limitations are noteworthy. Firstly, the use of the narrative review approach indicates reliance on gray literature sources and lack of critical appraisal of included studies. Thus, the conclusions reached need to be interpreted with caution. Secondly, we acknowledge that the term “gig nursing” is only an emerging terminology which may vary across settings. Also, considering the limited studies exploring gig nursing, it is challenging to attribute observed patient outcomes directly to platform-mediated staffing rather than broader temporary staffing arrangements.

## Conclusion

The gig economy's expansion into nursing presents both transformative potential and profound ethical and legal complexities. While gig work offers nurses unprecedented autonomy and flexibility, its unchecked growth risks normalizing precarious labor conditions, eroding workplace protections, and compromising care standards. To sustainably integrate this model, stakeholders must prioritize balance: preserving flexibility while embedding accountability. Policymakers must modernize labor laws to close classification loopholes; healthcare institutions should enforce rigorous standards for gig nurse engagement—including safe staffing ratios and credential verification—and platform developers must collaborate with regulators to ensure compliance with wage laws and scope-of-practice safeguards. By anchoring innovation in equity, transparency, and patient-centered values, the sector can harness the benefits of gig work without sacrificing the stability of the nursing workforce or the quality of care. The path forward demands not just adaptation, but a reimagining of labor frameworks that align twenty-first-century work models with timeless commitments to dignity, safety, and justice in healthcare.
